# Impact of the COVID-19 pandemic on knowledge, perceptions, and effects of telemedicine among the general population of Pakistan: A national survey

**DOI:** 10.3389/fpubh.2022.1036800

**Published:** 2023-01-05

**Authors:** Waleed Tariq, Muhammad Anas Tahseen Asar, Muhammad Junaid Tahir, Irfan Ullah, Qasid Ahmad, Ahmad Raza, Mohsin Khalid Qureshi, Ali Ahmed, Muhammad Zarak Sarwar, Muhammad Atif Ameer, Kaleem Ullah, Haziq Siddiqi, Muhammad Sohaib Asghar

**Affiliations:** ^1^Department of Medicine, Lahore General Hospital, Lahore, Pakistan; ^2^Department of Medicine, Kabir Medical College, Gandhara University, Peshawar, Pakistan; ^3^Department of Medicine, Mayo Hospital, Lahore, Pakistan; ^4^Department of Medicine, Hazrat Bari Imam Sarkar Medical and Dental College, Islamabad, Pakistan; ^5^Department of Medicine, School of Pharmacy, Monash University, Bandar Sunway, Malaysia; ^6^Department of Medicine, Shifa College of Medicine, Islamabad, Pakistan; ^7^Department of Medicine, Suburban Community Hospital, East Norriton, PA, United States; ^8^Department of Medicine, Pir Abdul Qadir Shah Jeelani Institute of Medical Sciences, Gambat, Pakistan; ^9^Department of Medicine, University of California, San Francisco, San Francisco, CA, United States; ^10^Department of Medicine, Mayo Clinic-Rochester, Rochester, MN, United States

**Keywords:** SARS-CoV-2, awareness, pandemic, telehealth, e-health, public health, COVID-19

## Abstract

**Background:**

Telemedicine is the provision of healthcare services through information and communication technology with the potential to mobilize all facets of the health sector to prevent the spread of COVID-19, provide quality healthcare, protect patients, doctors, and the public from exposure to disease, and reduce the burden on the healthcare system. This study aims to identify knowledge, perceptions, willingness to use, and the impact of the COVID-19 pandemic on telemedicine awareness.

**Methods:**

A cross-sectional study was conducted from 27 May 2020 to 17 June 2020 using the convenient sampling technique in the general population of Pakistan. Data were collected by designing an online questionnaire consisting of demographic information, knowledge, attitude perceptions, barriers, utilization, and the impact of the COVID-19 pandemic on telemedicine.

**Results:**

Of the 602 participants included in the study, 70.1% had heard about telemedicine, 54.3% had a good understanding of the definition of “telemedicine,” 81.4% had not used telemedicine in the past, 29.9% did not know that telemedicine was available before the COVID-19 pandemic, and 70.4% responded that the COVID-19 pandemic had changed their attitudes toward telemedicine. Gender (*p* = 0.017) and family income (*p* = 0.027) had a significant association with the perception of the benefits of telemedicine.

**Conclusion:**

The knowledge and usage of telemedicine are lacking due to inadequate awareness and technology. The need of the hour is to maximize the application of telemedicine to overcome the deficiencies of the healthcare system. Hence, it is essential to increase awareness through various means and develop an appropriate infrastructure to attain maximum benefits from telehealth services.

## Introduction

Information and communication technology (ICT) has revolutionized the provision of health and social care services in the form of e-health, telehealth, or telemedicine (1). According to the World Health Organization (WHO), telemedicine is the delivery of healthcare services to distant areas by healthcare professionals using ICT to exchange authentic information for the diagnosis, treatment, and prevention of illness and injuries, research and assessment, and training of healthcare providers, all in the interests of improving the healthcare services for the humanity (2).

Telemedicine assures quick doctor–patient interaction overcoming the barriers of distance and time. Scott et al. have reported that telemedicine could save an average distance of 145 miles and a time of 142 min per visit (3). Telemedicine has significantly reduced the cost of medical treatments and is also increasing access of the rural population to quality and specialized healthcare services immediately (4). Its scope is expanding beyond the management of chronic diseases and prescription compliance. The specialties in which its promising effects are seen include mental health, pediatrics, diabetes management, telemetry, cardiovascular treatments, retinopathy, nutrition trials, and hypertension (3, 5).

Telemedicine services have been proven effective in America, South-East Asia, and Europe (6). Its efficacy can be validated in underdeveloped regions suffering from a lack of medical staff, limited health facility centers, and vast distances (7). The doctor-to-patient ratio in Pakistan is much below the standard requirement (1.1 per 1,000), and the majority of the population (62.56%) lives in rural areas (8, 9). It has been acknowledged that telemedicine can increase the availability of healthcare services leading to improvement in the quality of healthcare in remote areas (5). Poor infrastructure, absence of technological expertise, and reduced awareness level of both doctors and patients are significant limitations in developing telemedicine in lower-middle-income countries (LMICS). Furthermore, socioeconomic status, confidentiality, reimbursement issues, privacy, legal concerns, resistance to change, and uncertain outcomes are essential obstacles that must be addressed (3).

Despite its role in chronic and common illnesses, telemedicine is also stepping toward emergency medical services, such as intensive care units (ICU), disaster management, and during epidemics (10). During the Ebola virus disease (EVD) outbreak, in 2014, epidemic countries such as Guinea, Liberia, Nigeria, and Indonesia utilized ICT for diagnosis and keeping track of the patient's health status and data collection (11, 12). Information collected was faster, secured, and a large number of patients were managed in less time. Telemedicine can also play a substantial role during the COVID-19 pandemic (7). The benefit of its use in epidemics is that it can prevent human-to-human transmission by reducing transportation and indoor clustering. Moreover, healthy people can be kept away from likely infected centers such as hospitals by remote screening, and also increases safe access to care for older people (13). Telemedicine has a significant role in medical consultation settings where direct physician–patient interaction is not required. Telemedicine can reduce the burden on resources, such as transportation, personal protective equipment (PPE), and the workload on healthcare providers (14). During the COVID-19 pandemic, telemedicine could help reduce hospital visits to avoid transmission, replace traditional medical visits, and facilitate patient and physician communication and cooperation (15). The implementation of telemedicine during the COVID-19 pandemic had proven to be feasible and effective with significant improvement in healthcare delivery, saving healthcare resources, reducing emergency hospital visits, and spread of COVID-19 (16, 17).

An essential factor in the implementation of telemedicine is the attitude of potential users. The willingness of patients to use depends on their attitude, satisfaction toward healthcare, and relationship with the healthcare providers (18). One of the primary concerns in using telemedicine is to have a secure mode of payment (19). Advances in secure money transfers such as online banking and micro-finance applications have made it easier for both consumers and providers to provide services (20). In this study, we used a willingness-to-pay (WTP) approach to find patients' preferences to repay telemedicine healthcare providers. The WTP approach was initially utilized in the environmental economics literature as a means to measure stated preferences for goods not sold in a marketplace. Still, it has been increasingly adopted as a measure to value healthcare options (21, 22).

Telemedicine was introduced in Pakistan in 1988 (23). But due to a lack of interest from government authorities, infrastructure, and technological shortfalls, it has not been used to its full potential. The legal policies regarding telemedicine are not available in the country (24). The awareness of telemedicine even among doctors in Pakistan is discouraging, as 63% were not familiar with telemedicine and 77% were not aware of any telemedicine programs available within the country, indicating the underdevelopment and low awareness level of telemedicine even among the healthcare providers in Pakistan (24). The literature regarding the awareness of telemedicine among the general population of Pakistan before the COVID-19 pandemic is lacking. The implementation of telemedicine in gastroenterology for outpatient practice during the COVID-19 pandemic had proclaimed satisfaction with the doctor–patient relationship (80%) and effectiveness in saving cost and time (85%) (25). The lack of regulatory framework in most countries is a significant drawback to integrating telemedicine services and the COVID-19 pandemic calls for the implementation of the necessary framework for telemedicine services (26). A systematic review from 2022 on telemedicine in Pakistan commented that a primary issue is the limited number of existing research studies (27). To the best of our knowledge, no previous study had been reported regarding awareness of telemedicine in the general population of Pakistan. Therefore, we conducted this study among the general population of Pakistan to find the level of awareness and utilization of telemedicine, the impact of the COVID-19 pandemic on the knowledge and attitude toward telemedicine, and the willingness to utilize and pay for telemedicine services in future.

## Methods and materials

### Study design and study setting

A cross-sectional, web-based study was conducted among the general population of Pakistan, and data were collected from 27 May 2020 to 17 June 2020.

### Ethical approval and participant consent

It was an observational study that valued the privacy and sovereignty of the participant, and personal information was kept anonymous. Participation was entirely voluntary, and participants could withdraw at any moment as per their choice. Electronic informed consent was obtained before initiating the survey. Ethical approval was granted by the honest review committee of Pir Abdul Qadir Shah Jeelani Institute of Medical Sciences, Gambat, Pakistan (Reference number: IRB/20/10).

### Sample size and sampling

A sample size of 601 was calculated using an online OpenEPI sample size calculator with a 95% confidence interval, 50% proportion of the population, and a 4% margin of error. The convenient sampling technique was used to collect data. The questionnaire was forwarded through WhatsApp sharing and multiple other social media platforms such as Facebook, Messenger, and Instagram. The incompletely filled questionnaires were not included. A total of 602 responses were included, with a response rate of 94%.

### Questionnaire development

The questionnaire was designed after a thorough literature review (3, 22, 28). It was composed in two languages, English and Urdu. The questionnaire was initially validated by experts in the medical profession and clinical research to assess the relativity and simplicity in accordance with the topic and population of this survey. Furthermore, a pilot study was conducted, and a questionnaire was distributed to a sample of 40 from the target populations, and participants were asked to provide feedback on instrument clarity and conciseness. Cronbach's alpha was reported to be 0.73. After face validation, one question was slightly modified based on the participants' comments, and the instrument's final version was developed. The proportion of samples used for face validation was not included in the primary study data.

The final questionnaire was comprised of six sections. The first section discussed demographic information, including age, gender, marital status, residence (urban and rural), education, and income. The second section was comprised of information regarding telemedicine, including prior knowledge, definition, and source of information. In the third section, we asked about telemedicine use in the past and the willingness to use it in the future. In the fourth section, we assessed the perception of participants about benefits (cost, saving of time, etc.) and barriers (lack of technology, mistrust of healthcare providers, etc.), which were graded on a Likert scale from 0 to 4 (0 = strongly disagree, 1 = disagree, 2 = neither disagree nor agree, 3 =agree, and 4 = strongly agree). The impact of the COVID-19 pandemic on the knowledge and attitude of people about telemedicine were components of the fifth section. The sixth section included questions that assessed the respondents' willingness to pay the telemedicine service fee in Pakistani rupee (PKR).

### Data analysis

First, data were entered in Microsoft Excel and subsequently imported into SPSS Version 22.0 for statistical analysis. Numerical variables were measured as mean and standard deviations, whereas categorical variables were expressed as frequencies and percentages. Inferential statistics were used, depending on the nature of the data and the variables. To find the non-random association between the groups, the normal distribution of the data was determined by the goodness-of-fit test and the chi-square test was applied further. A *p*-value of < 0.05 (two-tailed) was considered significant.

## Results

A total of 602 participants were included, with 323 (53.7%) male participants and 279 (46.3%) female participants. Approximately 507 (84.2%) belonged to the age group 20–29 years, 471 (78.2%) to the urban areas, 383 (56.6%) were undergraduates, and 165 (27.4%) had family income in the range of PKR 30,000–60,000 (USD 135–270) (Table 1).

**Table 1:** Sociodemographic characteristics of study participants (*N* = 602).

**Characteristic**	**Male *n* (%)**	**Female *n* (%)**
**Age**		
10–19	15 (2.5)	23 (3.8)
20–29	274 (44.5)	233 (38.7)
30–39	29 (4.8)	15 (2.5)
>39	5 (0.9)	8 (1.3)
**Marital status**		
Single	263 (43.7)	228 (37.9)
Married	60 (10.0)	51 (8.5)
**Residence**		
Rural	99 (16.5)	32 (5.3)
Urban	224 (37.2)	247 (41.0)
**Education**		
No formal	05 (0.8)	05 (0.8)
Primary	04 (0.7)	02 (0.3)
Secondary	10 (1.7)	07 (1.2)
Higher secondary	56 (9.3)	46 (7.6)
Undergraduate	203 (33.7)	180 (22.9)
Postgraduate	45 (7.5)	39 (6.5)
**Family income in PKR (USD)**		
< 30,000 (135)	65 (10.8)	33 (5.5)
30,000–60,000 (135–270)	83 (13.8)	82 (13.6)
60,000–90,000 (270–405)	60 (10.0)	55 (9.1)
90,000–120,000 (405–540)	46 (7.6)	42 (7.0)
>120,000 (540)	69 (11.5)	67 (11.1)

Regarding knowledge, 422 (70.1%) had heard about telemedicine, 254 (54.98%) had social media as a source of information about telemedicine, and 327 (54.3%) had a good understanding of the definition of “telemedicine.” A total of 180 participants (29.9%) did not know that telemedicine was available in Pakistan before the COVID-19 pandemic; only 77 (12.8%) responded that it could be used only during the COVID-19 pandemic, while 402 (66.8%) answered that it would be practiced in the future after the COVID-19 pandemic (Table 2).

**Table 2:** Knowledge and impact of COVID-19 on knowledge related to telemedicine service.

**Parameters**	***N* (%)**
**Have you ever heard about telemedicine?(*****N*** = **602)**	
Yes	422 (70.1)
No	180 (29.9)
**Wherefrom did you hear about telemedicine?**^a^ **(*****N*** = **462)**	
Television	39 (8.44)
Social media	254 (54.98)
Family and friend	116 (25.11)
Others	53 (11.47)
**What is your understanding of telemedicine?(*****N*** = **602)**	
Remote diagnosis and treatment of patients using telecommunications technology	327 (54.3)
Patients using the internet for their symptoms	18 (3.0)
Emailing reports of patients who cannot approach the doctor	16 (2.7)
Talking of doctor and patient through phone and text to get treatment	126 (20.9)
Don't know about telemedicine	115 (19.1)
**Was telemedicine available in Pakistan** **before COVID-19?(*****N*** = **602)**	
No	154 (25.6)
Yes	108 (17.9)
Maybe	160 (26.6)
Don‘t know	180 (29.9)
**Could telemedicine be used only during COVID-19?** **(*****N*** = **602)**	
No	373 (62.0)
Yes	77 (12.8)
Maybe	152 (25.2)
**Would telemedicine be practiced after COVID-19?(*****N*** = **602)**	
No	36 (6.0)
Yes	402 (66.8)
Maybe	164 (27.2)

^*a*^Single respondent may have opted for more than one option as checkbox options were given in the questionnaire.

Only 112 (18.6%) participants had used telemedicine, and 78 participants (18.6%) used it for common diseases like the common cold and fever. Out of 570 participants, 251 (44.1%) and 229 (40.2%) would use telemedicine for COVID-19 and common illnesses, respectively. Regarding attitudes, 262 (43.5%) participants responded to using telemedicine in the future and 251 (44.1%) will use it for COVID-19, and 424 (70.4%) had changed attitude toward telemedicine due to COVID-19 (Table 3).

**Table 3:** Practices and attitudes toward telemedicine.

**Questions**	***N* (%)**
**Practice and attitude**	
Practice: Have you ever used telemedicine?	*N* = 602
No	490 (81.4)
Yes	112 (18.6)
Practice: For what disease had you used telemedicine?^a^ (112 out of 602)	*N* = 112
For COVID-19	48 (42.9)
Common illness as common cold, fever, and headache, etc.	78 (69.7)
Chronic diseases as diabetes, high blood pressure, and joint pain, etc.	21 (18.8)
Serious illnesses/emergency conditions	4 (3.6)
Others	28 (25)
Attitude: Would you like to use telemedicine in the future?	*N* = 602
No	32 (5.3)
Yes	262 (43.5)
Maybe	308 (51.2)
Attitude: For what diseases would you like to use telemedicine?^a^ (570 out of 602)	*N* = 570
For COVID-19	251 (44.1)
Common illness as common cold, fever, and headache, etc.	229 (40.2)
Serious illnesses/emergency conditions	59 (10.4)
Chronic diseases as diabetes, high blood pressure, and joint pain, etc.	76 (13.4)
Other	62 (10.9)
**Impact of COVID-19 on attitude toward telemedicine**	
Has COVID-19 changed your attitude toward telemedicine?	*N* = 602
No	178 (29.6)
Yes	424 (70.4)
Do you think telemedicine would be practiced in long run in our society?	*N* = 602
No	72 (12.0)
Yes	297 (49.3)
Maybe	233 (38.7)

^*a*^Single respondent may have opted for more than one option as checkbox options were given in the questionnaire.

The perception of benefits and barriers regarding telemedicine was measured on a Likert scale with five answers ranging from strongly disagree (0) to strongly agreed (4). The majority of the participants had a positive perception toward telemedicine as it is cost-effective [264 (60.5%)], time-saving [445 (73.9%)], better for follow-up [430 (71.4%)], suitable for medical advice in rural areas [399 (66.2%)], and prevents unnecessary hospital visits [502 (83.4%)]. Regarding barriers, every disease could not be managed using telemedicine [415 (57.9%)], and it provides weak medical evidence for diagnosis [329 (54.6%)] (Table 4).

**Table 4:** Perception of benefits and barriers regarding telemedicine among participants (*N* = 602).

**Parameters**	**Strongly disagree *N* (%)**	**Disagree *N* (%)**	**Neither agree nor disagree *N* (%)**	**Agree (%)**	**Strongly agree (%)**	**Mean score**
**Perception regarding benefits of telemedicine** ^a^						
It is cost-effective	30 (5.0)	30 (5.0)	178 (29.6)	224 (37.2)	140 (23.3)	2.69
It is time-saving	20 (3.3)	22 (3.7)	115 (19.1)	244 (40.5)	201 (33.4)	2.97
Better for follow up	16 (2.7)	39 (6.5)	117 (19.4)	235 (39.0)	195 (32.4)	2.92
People from rural areas can get good medical advice	32 (5.3)	52 (8.6)	119 (19.8)	226 (37.5)	173 (28.7)	2.76
Can prevent unnecessary hospital visit	10 (1.7)	15 (2.5)	75 (12.5)	233 (38.7)	269 (44.7)	3.22
**Perception regarding barriers using telemedicine** ^a^						
Every disease cannot be managed	44 (7.3)	39 (6.5)	104 (17.3)	141 (13.4)	274 (44.5)	2.93
Due to lack of technology in Pakistan telemedicine cannot be practiced	41 (6.8)	89 (14.8)	189 (31.4)	175 (29.1)	108 (17.9)	2.37
Mistrust on technology	94 (15.6)	134 (22.3)	183 (30.4)	138 (22.9)	53 (8.8)	1.87
Mistrust on healthcare provider	132 (21.9)	146 (24.3)	156 (25.9)	122 (20.3)	46 (7.6)	1.67
Poor medical evidence for diagnosis	43 (7.1)	65 (10.8)	165 (27.4)	203 (33.7)	126 (20.9)	2.50

^*a*^Every question was answered on the Likert scale from 0 (strongly disagree) to 4 (strongly agree). A total score of 5 questions was in the range of 0–20.

Only 453 participants responded to section 6, that is WTP for telemedicine services. A total of 167 (36.8%) wanted “free” telemedicine service from a general practitioner, while 144 (31.7%) were willing to pay “PKR 500–1,000” (USD 2.25–4.50) for telemedicine service from a consultant (Table 5).

**Table 5:** Payment for telemedicine Service (PKR).

**Questions**	**Free, *N* (%)**	** < 500 (USD 2.25) (Rs/appointment), *N* (%)**	**500–1,000 (USD 2.25–4.5) (Rs/appointment), *N* (%)**	**1,000–1,500 (USD 4.5–6.75) (Rs/appointment), *N* (%)**	**>1,500 (USD 6.75) (Rs/appointment), *N* (%)**
How much would you like to pay telemedicine services to a general practitioner?(*N* = 453)	167 (36.8)	166 (36.6)	90 (19.8)	18 (3.9)	12 (8.0)
How much would you like to pay telemedicine services to a consultant? (*N* = 453)	125 (27.5)	107 (23.6)	144 (31.7)	55 (12.1)	22 (4.8)

There was a significant relationship between the male gender and good perception regarding the benefits of telemedicine (*p* = 0.017). Most of the participants belonging to urban areas (94.4%) had a bad perception regarding the benefits of telemedicine but this association was not statistically significant (*p* = 0.243). The family income (*p* = 0.027) had a statistically significant association with the perception of the benefits of telemedicine (Table 6).

**Table 6:** Association of perception regarding benefits of telemedicine with demographics (*N* = 602).

**Demography**	**Bad perception, ≤ 50% score^a^, *N* (%)**	**Good perception, >50% score^a^, *N* (%)**	***X* ^2^**	***p*-value**
**Age**			3.610	0.262
10–19	08 (21.1)	30 (78.9)		
20–29	66 (13.0)	441 (87.0)		
30–39	03 (6.8)	41 (93.2)		
>39	01 (7.7)	12 (92.3)		
**Gender**			5.747	0.017
Male	32 (9.9)	291 (90.1)		
Female	46 (16.5)	233 (83.5)		
**Marital status**			1.120	0.290
Single	67 (13.6)	424 (86.4)		
Married	11 (9.9)	100 (90.1)		
**Residence**			1.366	0.243
Rural	13 (9.9)	118 (90.1)		
Urban	65 (13.8)	406 (86.2)		
**Education**			3.322	0.614
No formal	1 (10.0)	9 (90.0)		
Primary	1 (16.7)	5 (83.3)		
Secondary	4 (23.5)	13 (76.5)		
Higher secondary	12 (11.8)	90 (88.2)		
Undergraduate	52 (13.6)	331 (86.4)		
Postgraduate	8 (9.5)	76 (90.5)		
**Family income in PKR (USD)**			10.927	0.027
< 30,000 (135)	18 (18.4)	80 (81.6)		
30,000–60,000 (135–270)	29 (17.6)	136 (82.4)		
60,000–90,000 (270–405)	8 (7.0)	107 (93.0)		
90,000–120,000 (405–540)	10 (11.4)	78 (88.6)		
>120,000 (540)	13 (9.6)	123 (90.4)		

^*a*^Five questions of perception regarding the benefits of telemedicine were answered on the Likert scale from 0 (strongly disagree) to 4 (strongly agree). A total score of five questions was in the range of 0–20, and we set a 50% cut-off point. The good perception was above 10 points, whereas the bad perception was 10 or fewer points.

## Discussion

Telemedicine is an emerging modality in healthcare that has proven beneficial to traditional medicine, in some ways, as it provides long-distance and widespread coverage in less time with fewer resources. The study was conducted to assess the impact of the COVID-19 pandemic on the knowledge of the general population about telemedicine in Pakistan.

In total, 70.1% of participants have awareness about telemedicine, being higher than reported by Arize et al. in southeast Nigeria, that is, 58.9% (22). Pazcka-Giorgi et al. found that 47.5% of lay persons had a moderate awareness level about telemedicine in Mexico (29). The higher awareness level in our study may be attributed to the high proportion of our participants belonging to urban areas (78.2%) and having completed some degree of post-secondary education (86.5%). Furthermore, the COVID-19 pandemic has raised awareness about telemedicine among the general population as they are depending more on telecommunication services for daily needs of healthcare and social services due to the lockdown and social distancing conditions in the country (7).

A total of 54.98% of participants responded that their source of information for telemedicine was social media, which was the same as anticipated in 59% of the world population using social media, which is why it has become a significant source of information for humans in this era (30). Social media campaigns had been effective in the promotion of health information because of their broad coverage, appeal, and cost-effectiveness (31).

In total, 18.6% of participants had already used telemedicine, and 43.5% were found willing to use it in the future. This is higher than a prior 2019 survey of young adults in Bulgaria, which found that 26% of young adults were willing to have a telemedicine consultation (32). The increased willingness may indicate increased public acceptance after COVID-19.

Regarding the attitude toward telemedicine, telemedicine was considered cost-effective (60.5%), time-saving (73.9%), prevents unnecessary hospital visits (83.4%), and better for follow-up (71.4%). Elhadi et al. reported that telemedicine could limit the spread of COVID-19 (94.8%), reduce hospital visits (93.6%), saves time by providing medical advice to remote areas (92.4%), and could facilitate patient and physician communication (91.5%) (15). Lack of transportation, cost of traveling, and long distances are barriers to healthcare services in underdeveloped regions, leading to higher direct and indirect costs to both the patient and the healthcare system. These factors usually lead to postponing or canceling the curative or preventive visit to the healthcare provider raising the burden on the hampered healthcare system, which can be elevated by promoting the application of telemedicine (33, 34).

In terms of perceived barriers, 56.5% of respondents were not assured of using telemedicine in the future as having the attitude toward the barriers that every disease could not be managed (57.9%) and the lack of technology is a hindrance in the use of telemedicine (47%). The lack of computer skills (21.9%), troubled transportation (20.5%), and poor internet connection (16.8%) are the self-reported barriers to telemedicine experience in rural China (35). Weißenfield et. al reported that false diagnosis (57%) and data privacy (31%) are the major risks of telemedicine implementation from the viewpoint of local government (36). The lack of access to internet services is the major factor hindering the development of telemedicine health delivery systems in Pakistan as only 36.5% population uses the internet (37). Furthermore, lack of knowledge, training of patients and staff, inadequate infrastructure, financial burden, and resistance by healthcare professionals are the other significant factors for the lack of a telemedicine healthcare delivery system (36, 38).

Only 17.9% of participants were aware that telemedicine was available in Pakistan before the COVID-19 pandemic, and 34.2% were not assured that telemedicine could be practiced after the COVID-19 pandemic. This highlights the low level of awareness in the community regarding the availability of healthcare services and the potential benefits of telemedicine. Pakistan is an LMIC and had health expenditure of only 3.3% of GDP, ultimately resulting in poor quality of healthcare services and awareness levels among the vast majority of the population in Pakistan (39).

Out of 453 participants, 63.2% and 72.5% were WTP for telemedicine to a general practitioner and consultant, respectively. Arize et al. reported that only 48.7% of respondents were WTP for telemedicine (22). The factors that affect the attitude of people toward WTP include unawareness, general cost, availability of technology, and socioeconomic status (3, 22). Another perspective was to find out how much people would pay for telemedicine. Socioeconomic status plays an important role in the healthcare disparity in low socioeconomic countries. The responses pertain to people not liking to pay more for telemedicine if they are consulting a specialist rather than a general practitioner.

## Implications

This study is the first of its kind to assess the perspectives of the general population in Pakistan about telemedicine. While most participants are interested in using telemedicine, they have concerns about the technology as well as limited knowledge about telemedicine availability. The specific concerns we identified can help guide health policymakers to make effective policies to implement telemedicine services in the region. Based on our findings about participants' sources of telemedicine information, it appears that targeted social media campaigns may be a particularly effective method for increasing awareness about telemedicine.

## Strengths and limitations

The strength of this study is that it has focused on an area of research where not much work has been done since the COVID-19 pandemic in Pakistan. However, our study had several limitations as follows: First, the convenient sampling method was used, and due to voluntary participation, there was a possibility of selection bias. Specifically, because this was an online survey, only people who already had internet access could participate, and we likely overrepresented people who are comfortable with using online services. Second, our sample size was more educated, wealthier, and more urban than the general population of Pakistan. Third, there may be recall bias in this study, particularly questions about prior telemedicine use. Finally, it is a self-reported study and though the responses were anonymous, participants may have felt pressured to conform to socially expected positive attitudes and perceptions about telemedicine. This study calls for additional research to focus on the underprivileged population with low education levels and limited asses to telecommunication services in underdeveloped regions, particularly since they may be the major expected beneficiary of telemedicine services.

## Conclusion

We found that the general population of Pakistan has some familiarity with telemedicine and a positive overall perception of telemedicine, but with poor knowledge of its utilization and availability in the country. The general population has positive thoughts about the time-saving capability and prevention of unnecessary transportation but expresses concern about telemedicine's versatility and susceptibility to technical issues. Because we found a limited understanding of telemedicine among our respondents despite their relatively higher education status, this study highlights the need to raise awareness regarding telemedicine in Pakistan. Participants were aware of the deficiencies that are required to overcome for the establishment and reimbursement of the infrastructure of telemedicine in the country. The awareness campaigns should be carried out on a massive scale through electronic, social, and print media. At a national level, a robust and efficient ICT infrastructure should be developed in the country for which substantial financial investments are imperative. Authorities should recognize the immediate need and take an interest to fulfill the rising demands of healthcare facilities by promoting telemedicine with the ultimate goal of improving healthcare for all.

## Data availability statement

The original contributions presented in the study are included in the article/supplementary material, further inquiries can be directed to the corresponding author.

## Ethics statement

The studies involving human participants were reviewed and approved by Pir Abdul Qadir Shah Jeelani Institute of Medical Sciences, Gambat, Pakistan (Reference number: IRB/20/10). The patients/participants provided their written informed consent to participate in this study.

## Author contributions

MT and IU conceived the idea. QA, AR, WT, MATA, and MT collected the data. IU and MSA analyzed and interpreted the data. WT, MATA, MT, MS, MAA, MSA, HS, HS, and AA wrote the manuscript. AA, KU, MSA, MQ, and IU critically reviewed the manuscript for intellectual content. All authors approved the final version of the manuscript.
